# Supplementary material for: Anatomical variability, multi-modal coordinate systems, and precision targeting in the marmoset brain
Source: Neuroimage. Author manuscript; Available in PMC 2022 Apr 15. (PMC8948178; doi:10.1016/j.neuroimage.2022.118965)
Supplement: 1 [file NIHMS1785795-supplement-1.docx]

**Supplementary Information**

**Anatomical variability, multi-modal coordinate systems, and precision targeting in the marmoset brain**

**Authors**

Takayuki Ose^1,2^, Joonas A. Autio^1^, Masahiro Ohno^1^, Stephen Frey^3^, Akiko Uematsu^1^, Akihiro Kawasaki^1^, Chiho Takeda^1^, Yuki Hori^1,4^, Kantaro Nishigori^1,5^, Tomokazu Nakako^1,5^, Chihiro Yokoyama^1,6^, Hidetaka Nagata^5^, Tetsuo Yamamori^7^, David C. Van Essen^8^, Matthew F. Glasser^8,9^, Hiroshi Watabe^2^, Takuya Hayashi^1,10^

**Affiliations**

^1^Laboratory for Brain Connectomics Imaging, RIKEN Center for Biosystems Dynamics Research, Kobe, Japan

^2^Graduate School of Biomedical Engineering, Tohoku University, Sendai, Japan

^3^Rogue Research Inc, Montreal, Canada

^4^Department of Functional Brain Imaging, National Institutes for Quantum and Radiological Science and Technology, Chiba, Japan

^5^Sumitomo Dainippon Pharma Co., Ltd., Osaka, Japan

^6^Faculty of Human life and Environmental Science, Nara women's University, Nara, Japan

^7^Laboratory for Molecular Analysis of Higher Brain Function, RIKEN Center for Brain Science, Wako, Japan

^8^Department of Neuroscience, Washington University Medical School, St Louis, MO USA

^9^Department of Radiology, Washington University Medical School, St Louis, MO USA

^10^Department of Brain Connectomics, Kyoto University Graduate School of Medicine, Kyoto, Japan

Incl. **Supplementary Figure S1, S2**


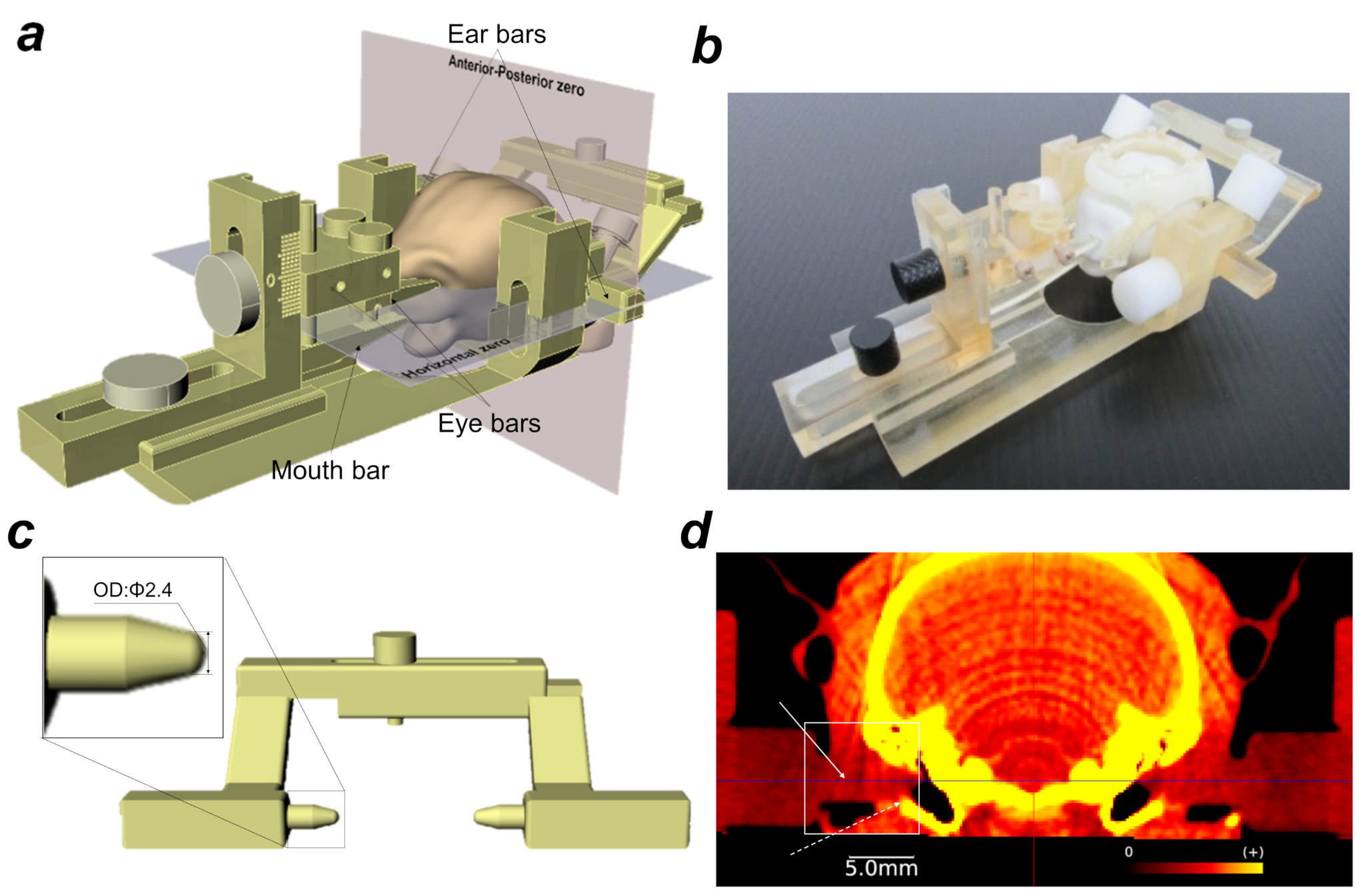


**Supplemental Figure 1.** Design of stereotactic device (a) The device was designed to be compatible with small animal CT (FOV = diameter 73 mm × height 57 mm). The head of the marmoset is mounted on the stereotactic device using mouth, eye and ear bars. (b) The marmoset phantom and head holder are firmly attached to the stereotactic device. (c, d) The tip of the ear bar matched the marmoset's auditory canal. (d) Coronal CT image. Solid and dashed arrows indicate the ear bars and the external auditory canals, respectively.


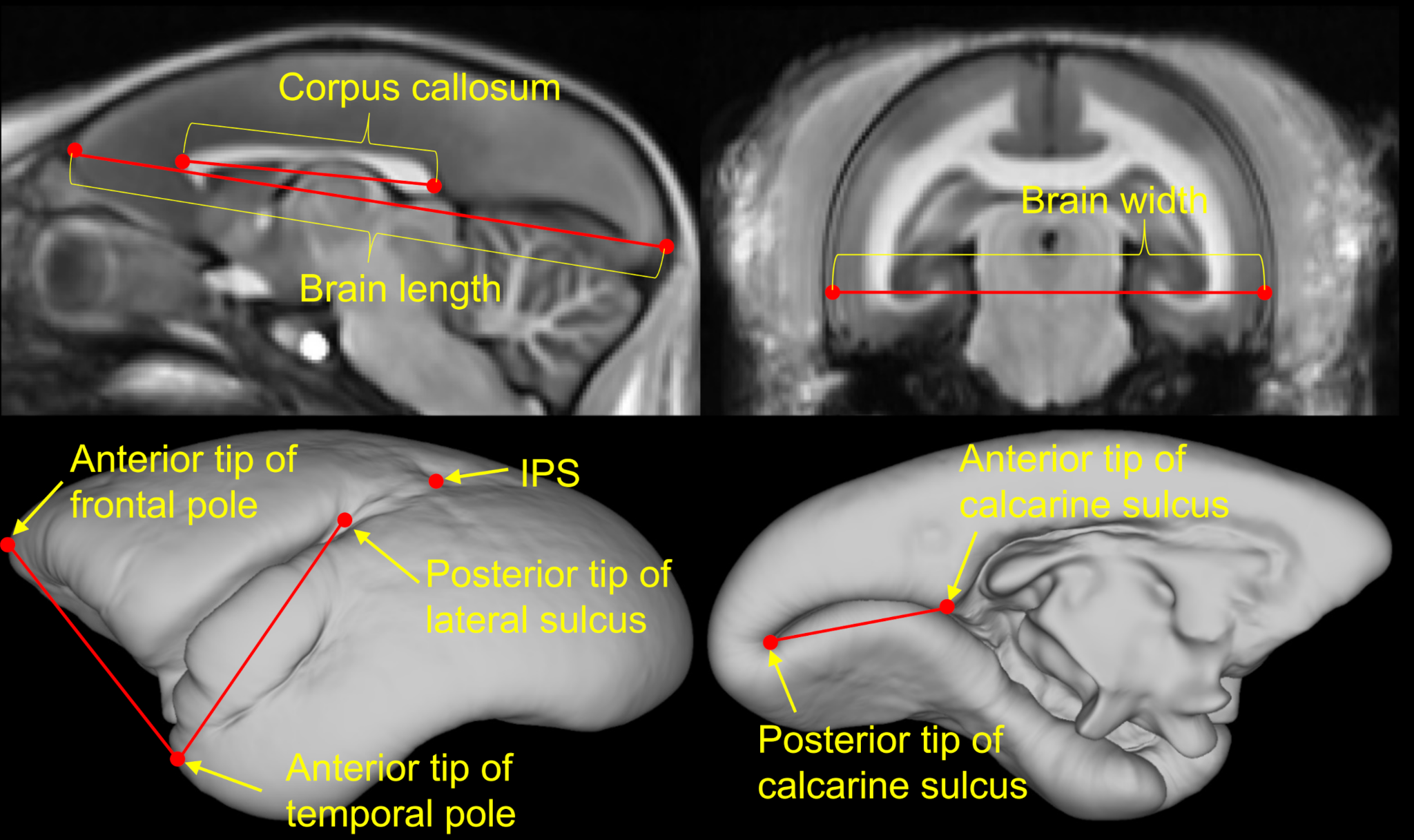


**Supplementary Figure 2**

The figure shows a schema of landmarks and distances of interest. The coordinates of landmarks were identified in T1w AC-PC native space and the Euclidean distances were measured in each animal.
